# A Comparison of Gene Set Analysis Methods in Terms of Sensitivity, Prioritization and Specificity

**DOI:** 10.1371/journal.pone.0079217

**Published:** 2013-11-15

**Authors:** Adi L. Tarca, Gaurav Bhatti, Roberto Romero

**Affiliations:** 1 Department of Computer Science, Wayne State University, Detroit, Michigan, United States of America; 2 Perinatology Research Branch, National Institute of Child Health and Human Development/National Institutes of Health, Rockville, Maryland, United States of America; 3 Department of Obstetrics and Gynecology, University of Michigan, Ann Arbor, Michigan, United States of America; 4 Department of Epidemiology and Biostatistics, Michigan State University, East Lansing, Michigan, United States of America; The University of Chicago, United States of America

## Abstract

Identification of functional sets of genes associated with conditions of interest from omics data was first reported in 1999, and since, a plethora of enrichment methods were published for systematic analysis of gene sets collections including Gene Ontology and biological pathways. Despite their widespread usage in reducing the complexity of omics experiment results, their performance is poorly understood. Leveraging the existence of disease specific gene sets in KEGG and Metacore® databases, we compared the performance of sixteen methods under relaxed assumptions while using 42 real datasets (over 1,400 samples). Most of the methods ranked high the gene sets designed for specific diseases whenever samples from affected individuals were compared against controls via microarrays. The top methods for gene set prioritization were different from the top ones in terms of sensitivity, and four of the sixteen methods had large false positives rates assessed by permuting the phenotype of the samples. The best overall methods among those that generated reasonably low false positive rates, when permuting phenotypes, were PLAGE, GLOBALTEST, and PADOG. The best method in the category that generated higher than expected false positives was MRGSE.

## Introduction

As soon as microarrays became available [1], scientists faced the challenge of interpreting the high volume of data generated from these technologies, as a typical experiment comparing two groups of samples can result easily in hundreds or thousands of genes being identified as differentially expressed between groups. One of the few options available to researchers to extracting meaning from such long lists of differentially expressed genes is to characterize the phenotype under the study by identifying over-represented/enriched categories of genes that share a similar function within the list of differentially expressed genes [2]. Even when a high-throughput experiment fails to demonstrate significant changes at gene level, due for instance to a modest effect or small sample size which are common in the field, gene set analysis is still relevant. This is because certain gene set analysis methods can use modest but coordinated changes in expression to establish a link between the phenotype and a predefined group of functionally related genes. As an example, Mootha et al. [3] showed that a set of genes involved in oxidative phosphorylation are coordinately decreased in the human diabetic muscle. A third application of gene set analysis methods is to compare gene expression changes across laboratories or even species. For instance, in the Species Translation Challenge (https://www.sbvimprover.com), a large international effort for systems biology verification, the effect of various stimuli on the transcriptome was expected to be translatable in a certain proportion between rat and human organisms, at gene set level rather than at the individual gene level.

The earliest approach [2] used to identify a common thread through the observed gene expression changes by finding over-represented/enriched categories of molecules that shared a similar function is the Over-Representation Analysis (ORA). This method became popular once software tools were designed to mine existing gene annotation databases including Gene Ontology [4], biological pathways databases (e.g. KEGG [5], Reactome [6]) and other gene set collections (e.g. MSigDB [7]). Over-representation approaches rely on a basic contingency table analysis testing for the association between the Differential Expression (DE) status of a gene (DE or not) and its membership in a given gene category (e.g. the set of genes related to apoptosis). Typical distributions used to perform ORA include hypergeometric, chi-square (χ^2^), etc. and they are implemented in publically available tools such as Onto-Tools [8], [9], GOstat [10], GOstats [11], and DAVID [12], just to mention a few. The drawbacks of ORA approaches include the fact that they cannot be applied if no DE genes are found in a given study, for instance due to lack of statistical power, and that the analysis result depends on the threshold used to select the DE genes. Also, the common assumption behind the models used for ORA, such as independence between genes, is likely violated, potentially resulting in an inflated rate of false positive findings [13].

A second generation of methods, called Functional Class Scoring (FCS) methods, alleviates the need to select significant genes as a first step and provide a unique result for a given dataset. Typically, these approaches derive a score from all genes that belong to a given gene set regardless of whether or not they are differentially expressed. Such methods include Gene Set Enrichment Analysis (GSEA) [7], GLOBALTEST [14], SIGPATHWAY [15] with two alternative null hypotheses (Q1 and Q2), Gene Set Analysis (GSA) [16], Generally Applicable Gene set Enrichment (GAGE) [17], Significance Analysis of Functional categories in gene Expression studies (SAFE) [18], Mean-Rank Gene Set Enrichment tests (MRGSE) [19], Pathway Analysis with Down-weighting of Overlapping Genes (PADOG) [20] that we have proposed previously, and Correlation Adjusted Mean Rank gene set test (CAMERA) [21].

A distinct type of gene set analysis methods compute a gene set score in each individual sample from the observed gene expression levels, and hence are deemed Single-Sample (SS) methods. The association between the phenotype and the sample-level gene set scores can be conducted with classical statistical models. This is an important advantage over FCS methods, because very complex designs (e.g. time series, longitudinal designs, etc.) can be easily analyzed in this way, while adjusting for relevant covariates in the analysis. The methods in this category that we considered in this work were: Pathway Level Analysis of Gene Expression (PLAGE) [22], Z-score [23], Single Sample GSEA (SSGSEA) [24] and Gene Set Variation Analysis (GSVA) [25]. See the Methods section for a very brief description of these approaches.

Among all methods described above there is a fundamental dichotomy with regard to their definition of the null hypothesis: *competitive methods* (ORA, MRGSE, GAGE, GSEAP, SIGPATHWAY-Q1), and *self-contained* methods [13] (all remaining methods mentioned above). From a practical standpoint, the competitive methods can be applied even with one sample per group as they rely on genes as sampling unit, yet they cannot work if no genes outside the gene set are measured. On the other hand, the self-contained methods use the subjects as the sampling unit and hence require several samples per group to infer significance of the gene sets. Unlike the competitive methods, some of the self-contained methods can be applied even when only the genes in the gene set are profiled. For a more detailed classification of gene set analysis methods see [26].

A separate class of analysis methods that exploit prior knowledge regarding the topology and gene-gene interactions available in biological pathways have also been proposed in the past [27], [28], and reviewed elsewhere [29]. Such methods are not within the scope of the current study for several reasons. Firstly, such methods were either designed for non-metabolic pathways only or for metabolic pathways only, while here we considered all KEGG metabolic and non-metabolic pathways. Secondly, for the Metacore Disease Biomarker Networks, the pathway information was not available in the format that these tools can use. Thirdly, our study is focused on gene set analysis methods which from a computational perspective have broader applicability than specialized pathway analysis methods, as they can be applied to the analysis of genes that form biological pathways as well as to any collection of custom defined gene sets.

Although the methods introduced above, and described in more detail in the Methods section, rely sometimes on a different null hypothesis and statistics, they basically assume that when a gene set is indeed relevant to a given phenotype, a sizeable proportion of the genes will show some amount of differential expression between groups in either one or both directions, depending on the method.

For the life scientist in need to occasionally perform a gene set analysis of his/her favorite dataset, as well as for the professional bioinformatician facing this task every day, it is difficult to know which approach works best because whenever these approaches were introduced, they were assessed in various ways using different gene sets collections, on few or no real datasets. Although methods for gene set analysis were recently reviewed [29] together with challenges in how to assess their performance, there is currently no large scale study that compares the performance of the existing approaches, and hence provide the community with a guide in selecting the best analysis method for this rather ubiquitous task.

The aim of this paper is therefore to provide a meaningful comparison of established gene set analysis methods in terms of their ability to i) rank close to the top gene sets that are indeed relevant to a given condition (*prioritization*), ii) produce small p-values for these relevant gene sets (*sensitivity*) while iii) not generating more false positives than expected (*specificity*). We relied on a scheme that uses particular KEGG and Metacore disease gene sets (e.g. Colorectal Cancer gene set in KEGG) and minimally assumed these to be relevant whenever a microarray datasets studying the corresponding phenotype (e.g. colorectal cancer vs normal) is used as input. A number of 42 such microarray datasets were selected from GEO for this goal totaling over 1,400 samples (see Table S1). The rank and p-value of one target gene set for each of the 42 datasets was the basis for assessing the prioritization ability and sensitivity of methods, while using permuted versions of these real datasets we determined their specificity with respect to the studied phenotype.

To make the comparison between the different methods fair, all methods used the data processed and filtered in the same way, and relied on identical gene set definitions. Moreover, to make the comparison between the sixteen different methods feasible when analyzing the large number of datasets we considered only methods that had software implementations suitable for batch operation implemented by the original authors or by others at a later point in time.

## Results

### Overall, the 42 Datasets Considered were a Good Match for the Diseases Studied; so were the KEGG/Metacore Gene Sets Specifically Designed for Those Diseases

**Figure 1:**
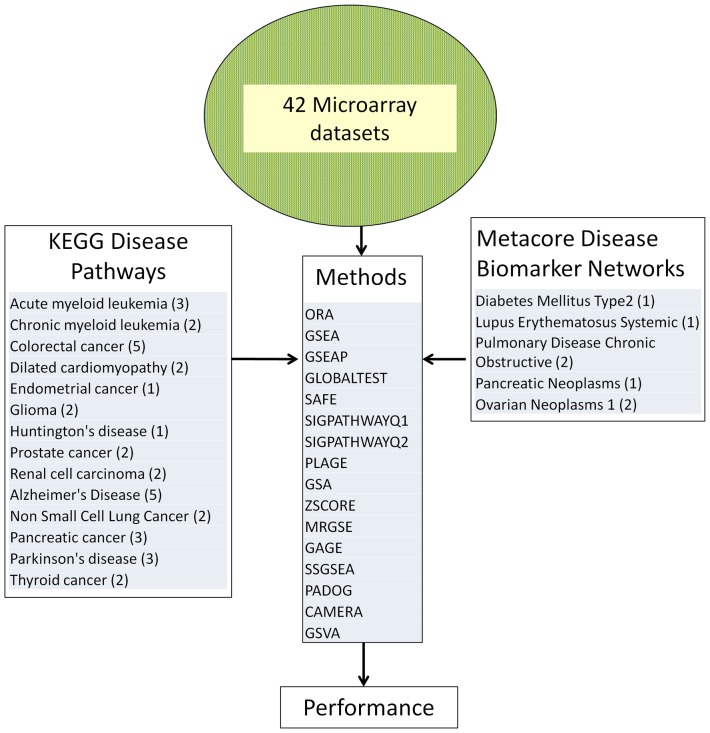
Procedure used to compare 16 gene set analysis methods. 42 microarray datasets were used, each studying a phenotype that has a corresponding KEGG or Metacore disease pathway, that we call target pathway. Each method was applied on each datasets and the p-value and rank of the target pathway in each dataset was used to compare the methods.

We applied sixteen different gene set analysis methods to analyze 259 KEGG gene sets and separately 88 Metacore® Disease Biomarker Networks. In each analysis, the input was one of the 42 independent public datasets that we selected and one of the two collections of gene sets (KEGG or Metacore). Each dataset studied one of 19 unique conditions/diseases shown in Figure 1, for which KEGG or Metacore has designed a specific gene set. We expected that in the output of each analysis method for a given dataset (sorting gene sets by p-value) the target gene set (having the same name as the condition under the study) will be ranked close to the top and have a small significance p-value.

**Figure 2:**
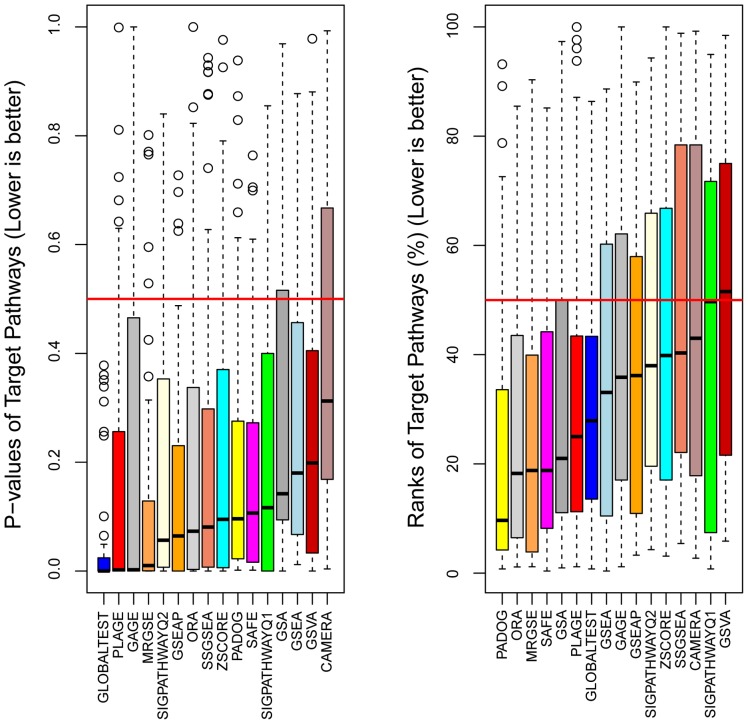
A comparison of sensitivity and prioritization ability of 16 gene set analysis methods. Each box contains 42 data points representing the p-value (left) and the rank (%) (right) that the target pathway received from a given method when using as input an independent dataset and a collection of gene sets (either KEGG or Metacore). Since the target pathways were designed by KEGG and Metacore for those diseases we expected that, in average, they will be found relevant by the different methods. Methods are ranked from best to worst according to the median p-value (left) and median rank (right).

Indeed, the median p-value of the target gene sets was significantly below 0.5 (Wilcoxon p<0.05) for all 16 methods studied (see Figure 2 left panel). Sorting the gene sets by their p-values within the output of each method and each dataset, we computed the rank of the target gene set as a percentage ranging from ∼0 to 100%. Small rank values (below 50%) would indicate that the target gene set was prioritized as relevant to the respective condition in a given dataset. Indeed, for 12 of the 16 methods the median rank was significantly lower than 50% (see Figure 2 right panel). These results suggest that overall a) the gene sets designed by KEGG and Metacore were relevant to those conditions, and b) that the datasets we selected captured the nature of those phenotypes, on average. An analysis of gene set analysis performance metrics in each of the 42 datasets separately revealed that 36 of the 42 datasets showed significant enrichment for the target gene set according to at least one method (false discovery rate <0.05 and rank <0.5) as shown in Figure S1.

### Most Sensitive Methods are not also the Best to Prioritize the Relevant Gene Sets

As a surrogate for the sensitivity of the methods, we used the median target gene set p-value over the 42 different datasets. The 16 methods were then ranked according to this metric (the smaller the median p-value the better the method). GLOBALTEST, PLAGE, GAGE and MRGSE were the top four methods (Figure 2 left panel). If instead of this surrogate for sensitivity, the classical definition of sensitivity is used with α = 0.05 for instance, the resulting ranking of the methods would be similar (see Table S2). We preferred though the median of p-values since it is threshold free and did not lead to ties in the ranking of the methods, unlike the classical definition of sensitivity (see Table S2). Since in this analysis we are only interested in the p-value of the target gene set in the output of a given method (one test per dataset), and since we are mainly interested in comparing the methods among them based on these p-values, no adjustment for multiple testing was necessary.

When the methods were sorted on the basis of the median rank of the target gene sets (a measure of prioritization ability of the methods), the top four methods were different, namely: PADOG, ORA, MRGSE and SAFE (Figure 2 right panel).

### Some Methods Find Significant Pathways Too Often Even in the Absence of Real Differential Expression

**Figure 3:**
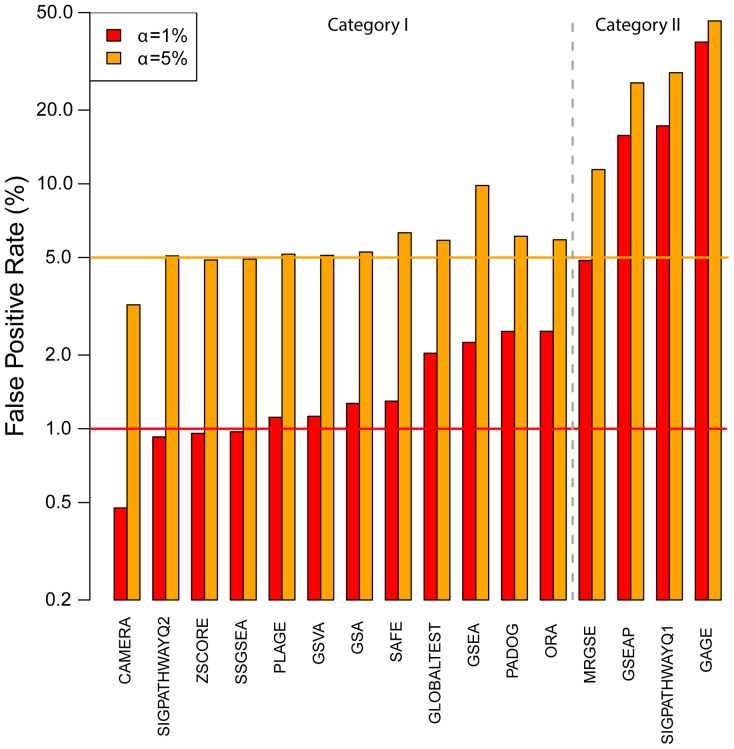
False positive rates produced by 16 gene sets analysis methods. The null hypothesis is simulated by analyzing phenotype permuted versions of the original datasets. The percentage of all pathways found significant at different significance levels (α) is reported for each method with a vertical bar. The horizontal lines denote the expected levels of false positives at each α level. Note the logarithmic scale.

Differences in the perceived sensitivity of the methods, as captured by the median p-values of the target gene sets in Figure 2 (left panel), can be due not only to an intrinsically higher sensitivity, but also to a higher than expected false positive rate. To determine the false positive rates in the absence of real differential expression, but presence of gene-gene correlations, we run the different methods on 50 phenotype permuted versions of each of the 42 datasets. We counted the number of gene sets with a p-value less than 0.01 and 0.05 under this null hypothesis and expressed this number as a percentage of the total number of tests. Figure 3 shows that four methods (all of the competitive type) namely GAGE, SIGPATHWAYQ1, MRGSE and GSEAP generated false positive rates that are much larger than the expected levels, unlike the remaining 12 methods. These four methods produced between 4.8 (MRGSE) and 37.9 (GAGE) times more false positives than expected at α = 1%. In contrast, all the other methods produced between 0.47% (CAMERA) and 2.5% (ORA) false positives.

### Plage, Globaltest and PADOG are the Best Overall Self-contained Methods; MRGSE the Best Competitive Method

To provide a meaningful overall ranking of the 16 methods considering their prioritization ability, sensitivity, and specificity, we produced two rankings, one for the methods that generated the expected (or close to expected) number of false positives under the permutation of the phenotype (category I), and one for the second category having much larger false positives rates under this null hypothesis (category II). Note that this dichotomization follows basically the competitive vs self-contained dichotomization except for ORA, which although is a competitive method produced a relatively low false positive rate. Overall, the mean false positive rate of methods in category I is 1.45% (SD = 0.68) while the best method in category II has significantly worse performance (p<0.0001) having a 4.9% false positive rate at α = 1%. After transforming the prioritization score and sensitivity surrogate (median target gene set p-values and median ranks respectively) into Z-scores (subtracting the median and dividing to the median of absolute deviations across the different methods in a given category) we have ranked the 12 methods in category I by the sum of their Z-scores. The best three methods that provide a good compromise between prioritization ability and sensitivity were PLAGE followed by GLOBALTEST and PADOG in category I. Using a similar approach we ranked the four methods in category II that have higher than expected false positive rates by also considering the amount of false positives that they generate in the ranking (see Table 1). The best of the four methods in this category was MRGSE as it was best in terms of prioritization ability and also in terms of false positive rates.

**Table 1:** Ranking of gene set analysis methods.

				Raw Data			Z-scores			Overall methodrank in category
Method	Sampling type	Category	Sensitivity	Prioritization	Specificity	Sensitivity	Prioritization	Specificity	Sum Z-scores	
			med. p	med. rank(%)	FP (α = 1%)	med. p	med. rank(%)	FP (α = 1%)		
**PLAGE**	subject	I	0.0022	25.0	1.1%	−1.5	−0.4	Not used	−1.86	1
**GLOBALTEST**	subject	I	0.0001	27.9	2.0%	−1.5	−0.2	Not used	−1.69	2
**PADOG**	subject	I	0.0960	9.7	2.5%	0.0	−1.5	Not used	−1.45	3
**ORA**	gene	I	0.0732	18.3	2.5%	−0.4	−0.9	Not used	−1.21	4
**SAFE**	subject	I	0.1065	18.8	1.3%	0.2	−0.8	Not used	−0.64	5
**SIGPATH. Q2**	subject	I	0.0565	38.0	0.9%	−0.6	−0.5	Not used	−0.09	6
**GSA**	subject	I	0.1420	21.0	1.3%	0.7	−0.7	Not used	0.07	7
**SSGSEA**	subject	I	0.0808	40.3	1.0%	−0.2	0.7	Not used	0.45	8
**ZSCORE**	subject	I	0.0950	39.8	1.0%	0.0	0.7	Not used	0.65	9
**GSEA**	subject	I	0.1801	33.1	2.3%	1.3	0.2	Not used	1.52	10
**GSVA**	subject	I	0.1986	51.5	1.1%	1.6	1.5	Not used	3.10	11
**CAMERA**	subject	I	0.3126	43.0	0.5%	3.4	0.9	Not used	4.30	12
**MRGSE**	gene	II	0.0100	18.8	4.9%	−0.59	−1.68	−1.27	−3.54	1
**GSEAP**	gene	II	0.0644	36.2	15.8%	0.59	0.02	−0.08	0.53	2
**GAGE**	gene	II	0.0024	35.9	37.9%	−0.76	−0.02	2.33	1.56	3
**SIGPATH. Q1**	gene	II	0.1165	49.7	17.2%	1.72	1.33	0.08	3.14	4

Surrogate sensitivity, prioritization ability and specificity are combined after transformation into Z-scores. A ranking is produced separately for methods in category I and methods in category II. Methods in category II produce substantially higher false positives than methods in category I under phenotype permutation.

We have evaluated the methods ranking stability as a function of several factors that could potentially impact the gene set analysis in different ways, such as the sample size of the microarray datasets, the gene set size, the type of experiment design, and the effect size of the condition under the study. The resulting rankings in the 8 scenarios shown in Table 2 were correlated with the original ranking of the methods (based on all datasets), with the Spearman correlation ranging between 0.78 for large gene sets scenario to 0.98 (all p<0.0001) for unpaired design scenario. The exception to the rule was the paired design scenario for which a 0.34 correlation coefficient was observed with the original ranking. Among the possible factors considered in Table 2, the sample size had the least effect on the methods ranking, with the correlation between the original ranking (based on 42 datasets) and the one based on the smallest and largest 21 datasets being 0.97 and 0.92 respectively (p<0.0001).

## Discussion

The value of gene set analysis is to reduce a potentially large list of differentially expressed genes (hundreds or a few thousands) into a smaller list of over-represented biological processes, molecular functions, biological pathways, etc. that can give a system’s level picture of the phenotype under the study. Also, even when differential expression cannot be claimed using usual stringency criteria, gene set analysis may still identify gene sets that are associated with a given phenotype by exploiting the fact that many genes in a gene set change with the condition under the study. The changes accumulated at gene set level can be either heterogeneous (both up- and down-regulation) or coordinated (same direction). Since infancy, the research in the field of gene set analysis was plagued with the lack of a gold standard. To measure sensitivity of gene set analysis, researchers have used either simulated data or, at most, a handful of real datasets in which prior knowledge was available regarding the relevance of some of the gene set to the considered phenotypes. In this work, we assumed only that some of the disease gene sets curated by KEGG and Metacore are relevant whenever the respective disease phenotype is studied using microarrays in an appropriate tissue/milieu. Since each dataset gives us just one performance measure data point we relied on an unprecedented number of GEO datasets (N = 42) for comparing gene sets analysis methods totaling over 1,400 samples. The assumption we relied on is not affected by the fact that there may be other gene sets at least as or more relevant to a given phenotype than the gene set that bears the same name as the condition under the study (that we call the target gene set). The main strength of this study is that we compared 16 methods on the same gene expression data and used the same collection of gene sets under the same conditions. The methods we considered were published in renowned peer reviewed international journals and have attracted a considerable number of citations (median of 25 citations per year) while spanning a period of 14 years.

The main findings of this study are:

- The use of multiple KEGG and Metacore curated disease gene sets together with a large number of datasets studying those respective diseases can be used as a benchmark for gene set analysis. This is because almost all of the 16 different methods showed evidence that the different gene sets were relevant to their respective conditions as captured by the median p-value and median rank of the target gene sets. In the absence of real differential expression (simulated by permuting the phenotype labels) the same gene sets appear as significant no more than expected by chance for 12 of the 16 methods. Although the KEGG and Metacore pathway databases were used to compare gene set analysis methods in this study, we acknowledge the fact that for identification of particular types of biological pathways (e.g. non-metabolic) other approaches that consider the pathway topology and gene interactions can be more suitable.
- The 42 datasets benchmark was made available to the community as the *KEGGdzPathwaysGEO* and *KEGGandMetacoreDzPathwaysGEO* R packages. The infrastructure required to run all 16 methods on these or any other dataset will be added to the development version of the Bioconductor *PADOG* package allowing therefore for reproducible research and easy comparison of new gene set analysis methods against existing ones.
- The best methods for gene set prioritization are different from the best methods in terms of sensitivity with PLAGE followed by GLOBALTEST and PADOG giving the best compromise between gene set prioritization and sensitivity while producing the expected rate of false positives whenever there are no real expression differences between groups. The most widely used gene set analysis method, GSEA (over 4500 citations according to Google Scholar), was ranked only 10^th^ of the 12^th^ methods that produce a rate of false positives close to the expected levels.
- As a point of interest, the three best overall methods are different in nature. GLOBALTEST tests if the variance of member gene coefficients in a logistic model is different from 0. PADOG combines differential expression moderated t-scores of the gene set members and weights them inversely to their frequency across gene sets. PLAGE computes one gene set score for each sample (the first principal component), and then scores can be tested for differences between groups using a moderated t-test.

The ranking of the methods that we propose in this work based on 42 datasets was consistent with the ranking based on only half of the datasets (Spearman rank correlation between 0.78 and 0.98, p<0.0001), with the dichotomy being established based on the total number of samples in each dataset, the target gene set size and the magnitude of changes between the conditions. When only the datasets with a paired design were considered, the ranking of the category II methods was identical with the one based on the overall data, yet, the ranking of category I methods was not correlated with the initial ranking based on all datasets. This is likely due to the rather small number of datasets with a paired design (n = 11). The discrepancy in the ranking stability between the two categories of methods can be understood at least in part by the fact that there are large differences between the category II methods according to the false positive criterion which induce stability in the ranking.

The results of this study confirm several previous findings, but contradict others. For instance, using analytic calculations and simulations Efron and Tibshirani [16] predicted that the GSA statistic (the maxmean) is generally more powerful than GSEA. Figure 2 shows that both in terms of sensitivity and ranking ability GSA performs better than GSEA, while both generate the same expected rate of false positives. Tarca et al. [20] have shown using 24 of the 42 datasets we used in this study that PADOG performs better than GSA and GSEA in analyzing KEGG gene sets, which is confirmed herein. Wu et al. [21] have shown that in the absence of real differential expression but small inter-gene correlation, the MRGSE method (a.k.a. geneSetTest in the *limma* package) will produce more false positives than expected, while their new method CAMERA does not show this drawback. Both these findings are replicated in Figure 3. Tomfohr et al. [22] suggest that their method PLAGE identifies gene sets that are more plausible than GSEA, a finding that is confirmed in Figure 2 (right panel) since PLAGE was ranked 6^th^ for gene set ranking ability while GSEA was ranked 8^th^.

In contrast, previous findings that suggest GSVA is superior to its single-sample enrichment counterparts ZSCORE, PLAGE and SSGSEA could not be replicated. Actually the opposite was true, as GSVA was the second to last ranked method. Luo et al. [17] claimed that their method, GAGE, outperforms GSEA in terms of the biological plausibility of the findings, which could not be replicated since GSEA produced better gene set ranking than GAGE. The perceived superiority of GAGE in terms of sensitivity is due to a very high false positive rate when there is no differential expression but gene correlations are present. The reason for this drawback of GAGE is that the method relies on gene sampling which, as for all gene sampling methods, results in smaller than expected p-values. In addition, by combining the p-values from multiple 1-on-group comparisons, yet assuming independence, the intrinsic lack of specificity of the method is amplified.

The goal of the gene set analysis benchmark presented in this study is to provide the reader with a guide to navigating the current landscape of existing methods and understand their strengths and limitations based on sensitivity, gene set prioritization, and specificity. Although a universally best gene set analysis method may still remain elusive, as is the case in other sub-fields of systems biology [30], in this work we provided a methods ranking considering one criterion at a time as well as a ranking considering combined criteria. If the user is mostly interested in defining a hypothesis regarding the involvement of certain functional groups of genes in a given condition, which can be later tested in a larger sample size, then choosing a method that gives best prioritization could be used, since such method is more likely to rank close to the top the meaningful gene sets. In this case the choice to be made will be between PADOG (if at least a handful of samples per group are available) or MRGSE and ORA if only 2–3 samples are available per group, or the experimental design is more complex (e.g. longitudinal design). If however, one is more interested in claiming significance of certain gene sets based on the available data, then GLOBALTEST would most likely give the best sensitivity for the test, although the significance p-values will not be ideal in establishing a ranking of the gene sets in relation with the phenotype. The best compromise method considering these two criteria is PLAGE, which also has slightly better specificity than GLOBALTEST and PADOG.

Some exceptions to this rather stable ranking based on all 42 datasets may be observed in Table 2, in which results obtained from half of the datasets would suggest that: i) GLOBALTEST is a better overall choice than PLAGE when the sample size is larger (over 22 samples in total) and that ii) ORA is the best overall method if the truly relevant gene sets are larger rather than smaller.

**Table 2:** Ranking of gene set analysis methods under several scenarios.

Method	Category	OverallRank	Sample size		Gene set size		Design		Effect	
			Smalln<22	Largen≥22	SmallN<66	LargeN≥66	Paired	Unpaired	Smallg<24.6%	Largeg≥24.6%
**PLAGE**	I	1	1	4	2	3	12	2	3	3
**GLOBALTEST**	I	2	2	1	3	5	6	1	2	4
**PADOG**	I	3	3	2	1	2	1	3	1	1
**ORA**	I	4	4	3	5	1	2	4	5	2
**SAFE**	I	5	7	5	4	8	8	5	4	6
**SIGPATH.Q2**	I	6	5	8	8	4	5	7	8	8
**GSA**	I	7	9	6	7	6	11	6	6	11
**SSGSEA**	I	8	8	7	6	12	4	8	9	5
**ZSCORE**	I	9	6	10	10	7	9	10	7	9
**GSEA**	I	10	10	9	9	11	10	9	10	7
**GSVA**	I	11	11	11	11	9	3	11	11	10
**CAMERA**	I	12	12	12	12	10	7	12	12	12
**MRGSE**	II	1	1	1	1	2	1	1	1	1
**GSEAP**	II	2	2	2	2	1	2	2	2	2
**GAGE**	II	3	3	3	3	4	3	3	3	3
**SIGPATH.Q1**	II	4	4	4	4	3	4	4	4	4

Datasets were divided into small sample size and large sample size using the median of sample sizes, 22, as cut-off. Similarly, the datasets were divided according to the number of genes in the target gene set using the median of gene set sizes, 66, as cut-off. Of the 42 experiments 11 were paired and 31 were not paired designs. To quantify the effect size, datasets were divided into a small effect group and a large effect group based on the % of genes with p<0.05, with the cut-of point being 24.6%.

In addition to the above described factors that could influence one’s choice of the method to use, we mention here that the context in which the methods are compared is one in which the gene sets considered are pathways instead of experimentally derived gene sets. In pathways, genes may be heterogeneously regulated in either direction whereas in experimentally derived gene sets, genes change in a coordinated fashion (mostly up- or down-regulated). The extent to which this aspect of the analysis has the potential to influence the ranking of the methods, some of which are specifically designed to detect coordinated changes, should be investigated further as gold standards are established for this purpose.

## Methods

### Sixteen Gene Set Analysis Methods

A brief introduction of the methods used in this study is given next: Gene Set Enrichment Analysis (GSEA) [7], [31] tests if the distribution of the ranks of genes in the gene set (ranking by p-values of association with the phenotype) differs from a uniform distribution using a weighted Kolmogorov-Smirnov test. An alternative version of GSEA that allows the user to define its own gene list ranking, and hence accommodate paired designs, is called GSEA Pre-Ranked (GSEAP), and is available from the same authors as GSEA. GSEAP does not rely on samples permutation as GSEA does, but it relies on gene sampling.

GLOBALTEST [14] uses a logistic regression model to determine if samples with similar profiles have similar phenotype by testing if the variance of the coefficients of genes in the gene set is different from 0. SIGPATHWAY of Tian et al. [15] tests two related yet distinct questions: i) the genes in a gene set show the same pattern of association with the phenotype compared to the rest of the genes (Q1) and ii) the gene set contains no DE genes (Q2). To detect moderate but coordinated associations for genes in a gene set (e.g. most genes are over expressed in disease than in control samples) a t-test is used to assess the shift in location of the gene correlations in the gene set of interest with respect to the background list of genes. In the same vein, Gene Set Analysis (GSA) [16] uses the *maxmean* statistic to determine if either up- or down-regulation of genes is the trend for which the evidence is the strongest for a particular gene set. While GSA is optimized to find gene sets with coordinated changes in one particular direction, Luo et al. [17] with their Generally Applicable Gene set Enrichment (GAGE) treat differently biological pathways from experimentally derived gene sets, since in pathways, genes may be heterogeneously regulated in either direction whereas in custom gene sets changes are usually coordinated. GAGE testes whether or not the mean fold changes of a target gene set is different from the one of the background list of genes profiled in the experiment. The method can therefore be applied even when only one sample per groups is available. When replicate samples exist, the resulting p-values from 1-on-1 comparisons are combined, assuming independence. Similar to GSEA and GSA, the Significance Analysis of Functional categories in Gene Expression studies (SAFE) [18] computes first a local gene level statistic (e.g. t-score) and then computes a gene set level statistic (i.e. Wilcoxon sum rank) to see if the distribution of local statistics in the gene set is different from the one of the background list of genes. Mean-Rank Gene Set Enrichment tests (MRGSE) [19] uses as null hypothesis that the given gene set is randomly chosen and tests if the ranks of genes in the genes et (sorted by a moderated t-test p-value) is different from the one of the background list. Pathway Analysis with Down-weighting of Overlapping Genes (PADOG) [20] was developed to account for the specificity of genes to certain gene sets and downplay the importance of the ubiquitous genes. The gene set score with PADOG is the mean of absolute moderated t-scores weighed inversely with the gene frequency across all gene sets analyzed. Correlation Adjusted Mean Rank gene set test (CAMERA) [21] method estimates the inter-gene correlation from data and uses it to adjust the gene set test statistic. To compute the significance p-value for the gene set summary statistics, FCS approaches described above rely either on sample (GSEA, GSA, SIGPATHWAY-Q2, SAFE, and PADOG) or gene (SIGPATHWAY-Q1, MRGSE) permutations. GLOBALTEST and GAGE rely on distributional assumptions. Pathway Level Analysis of Gene Expression (PLAGE) [22] works by decomposing the gene expression variance in each gene set by computing a meta-gene using singular value decomposition (SVD). This method is very much like using principal component analysis (PCA) to reduce gene expression dimensionality [32], by projecting the samples on the first principal component and discarding all remaining components. Lee et al. [23] summarized the gene set activity in a given sample using a Z-score (ZSCORE) after standardizing the gene expression levels across samples. Single Sample GSEA (SSGSEA) [24] calculates a sample level gene set score by comparing the distribution of gene expression ranks inside and outside the gene set. The Gene Set Variation Analysis (GSVA) [25] uses a non-parametric kernel to estimate the distribution of the gene expression level across all samples in order to bring the expression profiles to a common scale and then computes the Kolmogorov-Smirnov statistic similar to GSEA.

### Data Analysis

A total of 24 of the 42 microarray datasets used in this study, available from the Bioconductor’s *KEGGdzPathwaysGEO* package, were preprocessed using RMA as we previously described [20], while the additional 18 datasets were also either RMA normalized or obtained already normalized. The additional 18 datasets were made available as a new data package called *KEGGandMetacoreDzPathwaysGEO*. The datasets were produced using two different Affymetrix platforms that include multiple probesets for a given gene. Duplicate probesets per ENTREZ gene ID were removed by keeping the probeset with the highest average expression across all samples. KEGG pathways gene lists were obtained via the KEGGREST package (date stamp 4/22/2013) while the gene sets for the Metacore® Disease Biomarker Networks gene sets were downloaded from https://portal.genego.com (date stamp 4/19/2013).

The R packages *globaltest*, *gage*, *safe*, *sigPathway*, *GSVA*, *GSA*, and *PADOG*, were used to run the respective methods on every real or perturbed dataset. Implementations of ZSCORE, PLAGE and SSGSEA methods were available from the GSVA package. For the four methods that compute a gene set score per sample (ZSCORE, PLAGE, SSGSEA and GSVA), significance for the association with the disease was inferred using a paired or unpaired moderated t-test depending on the experimental design of each dataset. GSEA was run using GSEA.1.0.R function, while GSEAP analysis was performed using the java implementation (gsea2-2.0.12), both available from the Broad Institute website (http://www.broadinstitute.org/gsea). GSEAP was applied by ranking genes using a moderated [33] paired or unpaired t-test depending the study design of each dataset. For ORA analysis we implemented a one tailed hypergeometric test in R. The selection of DE genes for ORA was based on a moderated t-test p-value. The following strategy was used for gene selection for ORA: 1) use all genes with FDR [34] adjusted p-values<0.1 if more than 200; else go to next option; 2) use all genes with nominal p-values <0.05 and fold change>1.5 if more than 200; else go to next option; 3) Use top 1% of genes ranked by p-values. R version 3.0.1 was used for all analyses. A number of 1000 permutations were used in all analyses for the methods relying on sample or gene permutations including (GSA, GSEA, GSEAP, MRGSE, PADOG, SAFE, SIGPATHWAYQ1, SIGPATHWAYQ2).

The calculation of surrogate sensitivity, prioritization and specificity scores is illustrated in detail in Note S1.

## Supporting Information

Figure S1 Distribution of significance ranks (a) and p-values (b) obtained for the target pathway in each of the 42 datasets.
(DOCX)

Table S1 The 42 datasets used to compare the 16 gene set analysis methods.
(DOCX)

Table S2 Sensitivity of the pathway analysis methods at α = 0.01 and α = 0.05%.
(DOCX)

Note S1 Computation of surrogate sensitivity, prioritization and specificity from gene set analysis results.
(DOCX)

## References

[1] Schena M, Shalon D, Davis RW, Brown PO (1995) Quantitative monitoring of gene expression patterns with a complementary DNA microarray. Science 270: 467–470. 7569999 10.1126/science.270.5235.467

[2] Tavazoie S, Hughes JD, Campbell MJ, Cho RJ, Church GM (1999) Systematic determination of genetic network architecture. Nat Genet 22: 281–285. 10391217 10.1038/10343

[3] Mootha VK, Lindgren CM, Eriksson KF, Subramanian A, Sihag S, et al (2003) PGC-1alpha-responsive genes involved in oxidative phosphorylation are coordinately downregulated in human diabetes. Nat Genet 34: 267–273. 12808457 10.1038/ng1180

[4] Ashburner M, Ball CA, Blake JA, Botstein D, Butler H, et al (2000) Gene ontology: tool for the unification of biology. The Gene Ontology Consortium. Nat Genet 25: 25–29. 10802651 10.1038/75556 PMC3037419

[5] Ogata H, Goto S, Sato K, Fujibuchi W, Bono H, et al (1999) KEGG: Kyoto Encyclopedia of Genes and Genomes. Nucleic Acids Res 27: 29–34. 9847135 10.1093/nar/27.1.29 PMC148090

[6] Joshi-Tope G, Gillespie M, Vastrik I, D’Eustachio P, Schmidt E, et al (2005) Reactome: a knowledgebase of biological pathways. Nucleic Acids Res 33: D428–D432. 15608231 10.1093/nar/gki072 PMC540026

[7] Subramanian A, Tamayo P, Mootha VK, Mukherjee S, Ebert BL, et al (2005) Gene set enrichment analysis: a knowledge-based approach for interpreting genome-wide expression profiles. Proc Natl Acad Sci U S A 102: 15545–15550. 16199517 10.1073/pnas.0506580102 PMC1239896

[8] Khatri P, Draghici S, Ostermeier GC, Krawetz SA (2002) Profiling gene expression using onto-express. Genomics 79: 266–270. 11829497 10.1006/geno.2002.6698

[9] Draghici S, Khatri P, Martins RP, Ostermeier GC, Krawetz SA (2003) Global functional profiling of gene expression. Genomics 81: 98–104. 12620386 10.1016/s0888-7543(02)00021-6

[10] Beissbarth T, Speed TP (2004) GOstat: find statistically overrepresented Gene Ontologies within a group of genes. Bioinformatics 20: 1464–1465. 14962934 10.1093/bioinformatics/bth088

[11] Falcon S, Gentleman R (2007) Using GOstats to test gene lists for GO term association. Bioinformatics 23: 257–258. 17098774 10.1093/bioinformatics/btl567

[12] Huang DW, Sherman BT, Tan Q, Kir J, Liu D, et al (2007) DAVID Bioinformatics Resources: expanded annotation database and novel algorithms to better extract biology from large gene lists. Nucleic Acids Res 35: W169–W175. 17576678 10.1093/nar/gkm415 PMC1933169

[13] Goeman JJ, Buhlmann P (2007) Analyzing gene expression data in terms of gene sets: methodological issues. Bioinformatics 23: 980–987. 17303618 10.1093/bioinformatics/btm051

[14] Goeman JJ, van de Geer SA, de KF, van Houwelingen HC (2004) A global test for groups of genes: testing association with a clinical outcome. Bioinformatics 20: 93–99. 14693814 10.1093/bioinformatics/btg382

[15] Tian L, Greenberg SA, Kong SW, Altschuler J, Kohane IS, et al (2005) Discovering statistically significant pathways in expression profiling studies. Proc Natl Acad Sci U S A 102: 13544–13549. 16174746 10.1073/pnas.0506577102 PMC1200092

[16] Efron B, Tibshirani R (2007) On testing the significance of sets of genes. Ann Appl Stat 1: 107–129.

[17] Luo W, Friedman MS, Shedden K, Hankenson KD, Woolf PJ (2009) GAGE: generally applicable gene set enrichment for pathway analysis. BMC Bioinformatics 10: 161. 19473525 10.1186/1471-2105-10-161 PMC2696452

[18] Barry WT, Nobel AB, Wright FA (2005) Significance analysis of functional categories in gene expression studies: a structured permutation approach. Bioinformatics 21: 1943–1949. 15647293 10.1093/bioinformatics/bti260

[19] Michaud J, Simpson KM, Escher R, Buchet-Poyau K, Beissbarth T, et al (2008) Integrative analysis of RUNX1 downstream pathways and target genes. BMC Genomics 9: 363. 18671852 10.1186/1471-2164-9-363 PMC2529319

[20] Tarca AL, Draghici S, Bhatti G, Romero R (2012) Down-weighting overlapping genes improves gene set analysis. BMC Bioinformatics 13: 136. 22713124 10.1186/1471-2105-13-136 PMC3443069

[21] Wu D, Smyth GK (2012) Camera: a competitive gene set test accounting for inter-gene correlation. Nucleic Acids Res 40: e133. 22638577 10.1093/nar/gks461 PMC3458527

[22] Tomfohr J, Lu J, Kepler TB (2005) Pathway level analysis of gene expression using singular value decomposition. BMC Bioinformatics 6: 225. 16156896 10.1186/1471-2105-6-225 PMC1261155

[23] Lee E, Chuang HY, Kim JW, Ideker T, Lee D (2008) Inferring pathway activity toward precise disease classification. PLoS Comput Biol 4: e1000217. 18989396 10.1371/journal.pcbi.1000217 PMC2563693

[24] Barbie DA, Tamayo P, Boehm JS, Kim SY, Moody SE, et al (2009) Systematic RNA interference reveals that oncogenic KRAS-driven cancers require TBK1. Nature 462: 108–112. 19847166 10.1038/nature08460 PMC2783335

[25] Hanzelmann S, Castelo R, Guinney J (2013) GSVA: gene set variation analysis for microarray and RNA-Seq data. BMC Bioinformatics 14: 7. 23323831 10.1186/1471-2105-14-7 PMC3618321

[26] Maciejewski H (2013) Gene set analysis methods: statistical models and methodological differences. Brief Bioinform. 10.1093/bib/bbt002 PMC4103537 23413432

[27] Tarca AL, Draghici S, Khatri P, Hassan SS, Mittal P, et al (2009) A novel signaling pathway impact analysis. Bioinformatics 25: 75–82. 18990722 10.1093/bioinformatics/btn577 PMC2732297

[28] Draghici S, Khatri P, Tarca AL, Amin K, Done A, et al (2007) A systems biology approach for pathway level analysis. Genome Res 17: 1537–1545. 17785539 10.1101/gr.6202607 PMC1987343

[29] Khatri P, Sirota M, Butte AJ (2012) Ten years of pathway analysis: current approaches and outstanding challenges. PLoS Comput Biol 8: e1002375. 22383865 10.1371/journal.pcbi.1002375 PMC3285573

[30] Tarca AL, Lauria M, Unger M, Bilal E, Boue S, et al. (2013) Strengths and limitations of microarray-based phenotype prediction: Lessons learned from the IMPROVER Diagnostic Signature Challenge. Bioinformatics. 10.1093/bioinformatics/btt492 PMC3810846 23966112

[31] Mootha VK, Lindgren CM, Eriksson KF, Subramanian A, Sihag S, et al (2003) PGC-1alpha-responsive genes involved in oxidative phosphorylation are coordinately downregulated in human diabetes. Nat Genet 34: 267–273. 12808457 10.1038/ng1180

[32] Tarca AL, Carey VJ, Chen XW, Romero R, Draghici S (2007) Machine learning and its applications to biology. PLoS Comput Biol 3: e116. 17604446 10.1371/journal.pcbi.0030116 PMC1904382

[33] Smyth GK (2012) Limma: linear models for microarray data. In: Gentleman R, Carey VJ, Huber W, Irizarry RA, Dudoit S, editors. Bioinformatics and Computational Biology Solutions Using R and Bioconductor. Springer. 397–420.

[34] Benjamini Y, Hochberg Y (1995) Controlling the false discovery rate: a practical and powerful approach to multiple testing. J Royal Stat Soc B 57: 289–300.

