# Supplementary material for: A Comparison of Gene Set Analysis Methods in Terms of Sensitivity, Prioritization and Specificity
Source: PLoS One. 2013 Nov 15;8(11):e79217. doi: 10.1371/journal.pone.0079217 (PMC3829842; doi:10.1371/journal.pone.0079217)
Supplement: Figure S1 — Distribution of significance ranks (a) and p-values (b) obtained for the target pathway in each of the 42 datasets. (DOCX) [file pone.0079217.s001.docx]

Figure S1: Distribution of significance ranks (a) and p-values (b) obtained for the target pathway in each of the 42 datasets by the 16 analysis methods. Only 6 of the 42 datasets marked with a (-) sign did not show evidence that they were a good match for the phenotype studied and/or that the target pathway was suited for the phenotype. For the remaining 36 datasets at least one method produced a false discovery rate adjusted p-value <0.05 for the target pathway while at the same time ranking the target pathway in the upper half.

a
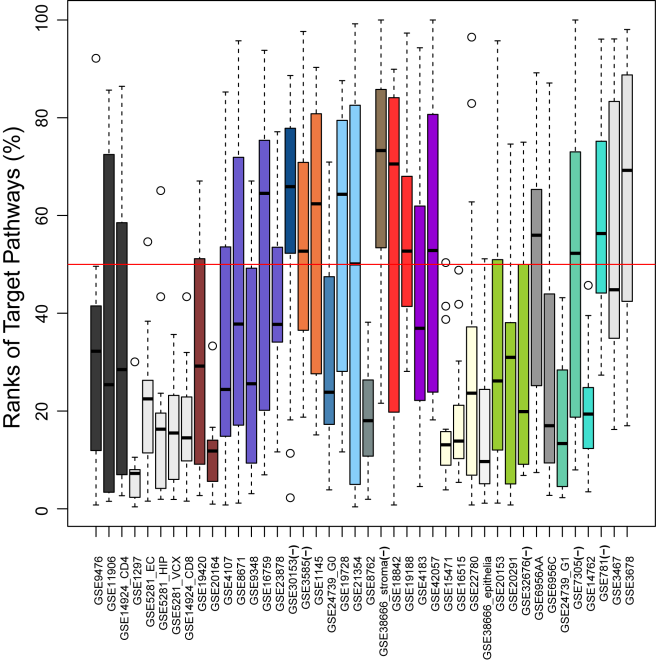

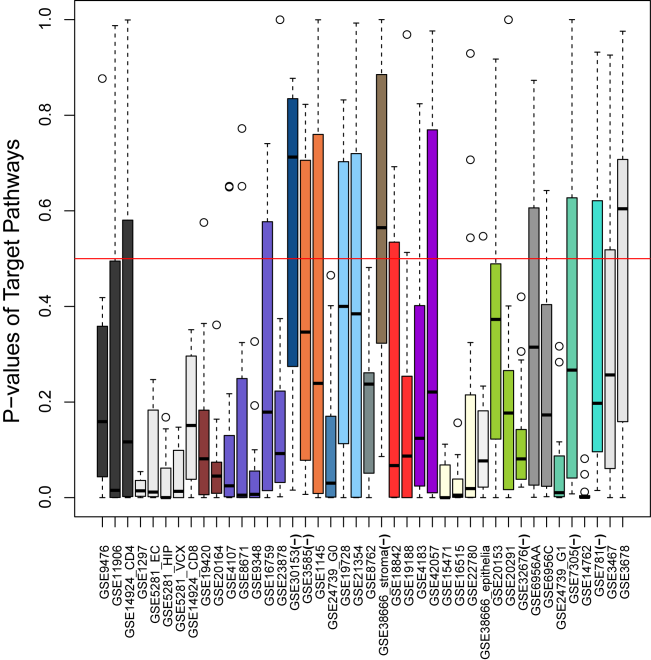


b
