# Supplementary material for: A Comparison of Gene Set Analysis Methods in Terms of Sensitivity, Prioritization and Specificity
Source: PLoS One. 2013 Nov 15;8(11):e79217. doi: 10.1371/journal.pone.0079217 (PMC3829842; doi:10.1371/journal.pone.0079217)
Supplement: Table S1 — The 42 datasets used to compare the 16 gene set analysis methods. (DOCX) [file pone.0079217.s002.docx]

Table S1: The 42 datasets used to compare the 16 gene set analysis methods. The name of pathway in the KEGG or Metacore database assumed to be relevant for a given dataset is the same as the name of the disease studied.
