# Supplementary material for: A Comparison of Gene Set Analysis Methods in Terms of Sensitivity, Prioritization and Specificity
Source: PLoS One. 2013 Nov 15;8(11):e79217. doi: 10.1371/journal.pone.0079217 (PMC3829842; doi:10.1371/journal.pone.0079217)
Supplement: Table S2 — Sensitivity of the pathway analysis methods at α = 0.01 and α = 0.05%. (DOCX) [file pone.0079217.s003.docx]

Table S2: Sensitivity of the pathway analysis methods at α=0.01 and α=0.05%. Note that multiple ties are obtained in the ranking if this classical definition of sensitivity is used instead of the surrogate sensitivity.

|  | α=0.01 | α=0.05 |
| --- | --- | --- |
| GLOBALTEST | 66.7 | 78.6 |
| GAGE | 59.5 | 64.3 |
| PLAGE | 59.5 | 64.3 |
| MRGSE | 50 | 61.9 |
| GSEAP | 35.7 | 50 |
| ORA | 35.7 | 47.6 |
| SIGPATHWAYQ2 | 28.6 | 45.2 |
| SSGSEA | 31 | 45.2 |
| PADOG | 16.7 | 40.5 |
| SAFE | 19 | 40.5 |
| SIGPATHWAYQ1 | 33.3 | 40.5 |
| ZSCORE | 26.2 | 40.5 |
| GSVA | 14.3 | 28.6 |
| GSA | 4.8 | 16.7 |
| GSEA | 0 | 14.3 |
| CAMERA | 2.4 | 4.8 |
