# Supplementary material for: A Comparison of Gene Set Analysis Methods in Terms of Sensitivity, Prioritization and Specificity
Source: PLoS One. 2013 Nov 15;8(11):e79217. doi: 10.1371/journal.pone.0079217 (PMC3829842; doi:10.1371/journal.pone.0079217)
Supplement: Note S1 — Computation of surrogate sensitivity, prioritization and specificity from gene set analysis results. (DOCX) [file pone.0079217.s004.docx]

Note S1: Computation of surrogate sensitivity, prioritization and specificity from gene set analysis results

*Surrogate sensitivity*

Assume for simplicity that we have only 2 datasets (instead of 42) and there are only 5 gene sets to analyze (instead of 259 for KEGG and 88 for Metacore), which we rank by p-value in each dataset separately for a given method. Let gene set B and A be the target gene set in Dataset 1 and 2 respectively.

| Dataset 1 | Target gene set is B | |  | Dataset 2 | Target gene set is A | |
| --- | --- | --- | --- | --- | --- | --- |
|  |  |  |  |  |  |  |
|  | p-values |  |  |  | p-values |  |
| E | 0.01 |  |  | A | 0.02 |  |
| B | 0.04 |  |  | C | 0.07 |  |
| C | 0.1 |  |  | D | 0.3 |  |
| D | 0.4 |  |  | B | 0.5 |  |
| A | 0.6 |  |  | E | 0.7 |  |

The surrogate sensitivity will be the median of the target pathway p-values, i.e. median of (0.04, 0.02) values, i.e. 0.03. The classical sensitivity in this case at α=0.05 would be 50% as, of the two tests expected to be positive (gene set B in dataset 1 and gene set A in dataset 2) only one met the threshold p<0.05 to be claimed positive, and hence counted as a true positive out of two positives.

*Prioritization*

To compute the prioritization score for this method we need to convert the p-values into ranks. The rank of the target gene set in Dataset 1 is 2 (since gene set B is the second row in the table) / 5*100=40%, while for dataset 2 it is 1/5*100=20%. The prioritization score will be the median of the two rank values (40%, and 20%), i.e. 30%.

*Specificity*

To illustrate the computation of specificity, assume that for the same setup described above, the analysis result of a given method is obtained on one (instead of 50) permuted version of 2 (instead of 42) datasets, as shown below:

| Permuted Dataset 1 | |  |  | Permuted Dataset 2 | |  |
| --- | --- | --- | --- | --- | --- | --- |
|  |  |  |  |  |  |  |
|  | p-values |  |  |  | p-values |  |
| A | 0.6 |  |  | A | 0.7 |  |
| B | 0.02 |  |  | B | 0.4 |  |
| C | 0.9 |  |  | C | 0.01 |  |
| D | 0.5 |  |  | D | 0.32 |  |
| E | 0.6 |  |  | E | 0.69 |  |

The false positive rate in this case at α=0.05 will be 2/10=20%, since 2 p-values of the 10 tests (2 datasets x 5 gene sets) are less than 0.05. The specificity of the method will be 1- false positive rate=80%.
